# Impediment of Cerebrospinal Fluid Drainage Through Glymphatic System in Glioma

**DOI:** 10.3389/fonc.2021.790821

**Published:** 2022-01-10

**Authors:** Dan Xu, Jie Zhou, Hao Mei, Huan Li, Wenbo Sun, Haibo Xu

**Affiliations:** Department of Radiology, Zhongnan Hospital of Wuhan University, Wuhan University, Wuhan, China

**Keywords:** cerebrospinal fluid (CSF), glymphatic system, perivascular space, aquaporin 4 (AQP4), glioma, intracisternal injection

## Abstract

**Background:**

Cerebrospinal fluid (CSF) plays an important role in maintaining tissue homeostasis in the central nervous system. In 2012, the new CSF outflow pathway, “the glymphatic system,” was discovered. The glymphatic system mediates CSF and interstitial fluid exchange through the perivascular pathway, which eliminates harmful solutes in the brain parenchyma. In recent studies, the importance of the glymphatic system has been demonstrated in healthy and neurodegenerative disease brains. However, there is limited research on the function of the CSF in brain tumors. Intracranial hypertension caused by glioma can affect CSF drainage, which impacts the delivery of chemotherapy drugs *via* intrathecal injection. This study focused on changes in the glymphatic system and the role of aquaporin 4 (AQP4) in glymphatic transport in glioma.

**Methods:**

In glioma-bearing rats, the effect of tracer infusion on the intracranial pressure (ICP) was evaluated using an ICP microsensor. *In vivo* magnetic resonance imaging and *ex vivo* bright field were used to monitor CSF tracer distribution after cisterna magna injection. AQP4 expression was quantitatively detected, and AQP4 in the astrocytes around the vessels was observed using immunofluorescence.

**Results:**

The ICP of the tumor group was higher than that of the control group and the infusion rate of 2 µl/min did not affect ICP. *In vivo* and *ex vivo* imaging showed that the circulation of CSF tracers was significantly impaired in the tumor. High-power confocal microscopy revealed that, in the tumor, the surrounding of AQP4 by Evans Blue was decreased. In both tumor and contralateral areas, data indicated that the number of cluster designation 34 (CD34^+^) alpha-smooth muscle actin (α-SMA^−^) veins were more than that of CD34^+^α-SMA^+^ arteries. Moreover, in the tumor area, AQP4 in the astrocytes around the vessels was decreased.

**Conclusions:**

These findings indicate that the para-arterial influx of subarachnoid CSF is limited in glioma, especially in those with reduced levels of the fundamental protein AQP4. Our results provide evidence toward a potential new treatment method for glioma in the future.

## Introduction

Cerebrospinal fluid (CSF) is produced by the choroid plexus in the ventricular system, which is formed by four interconnected cavities (two lateral ventricles and the third and fourth ventricles). The central nervous system (CNS) is unlike other organs and has no lymphatic vessels in the brain parenchyma, through which metabolites can be eliminated *via* the lymphatic system. Therefore, to maintain tissue homeostasis and its normal function, the CSF is thought to play a role in the clearance of solutes from the brain.

Traditional theories of CSF drainage suggest that CSF is generated by the ventricles and flows into the subarachnoid space, absorbed by the arachnoid particles and arachnoid villi, and carried to the venous sinuses (1–5). However, recent studies have revealed other CSF exit pathways (1): along the nerves, such as the olfactory and spinal nerves, leaving the skull to reach the cervical and spinal lymph nodes (2, 3, 6, 7); flowing out through the “glymphatic system” (8–13). In 2012, Iliff et al. (8) discovered the “glymphatic system” in the mouse brain for the first time. The glymphatic system is defined as a brain-wide perivascular pathway driven by astrocytic aquaporin 4 (AQP4). It plays an important role in clearing the interstitial solutes in the brain parenchyma and involves the para-arterial influx of subarachnoid CSF into the brain interstitium, the exchange of CSF with interstitial fluid (ISF), followed by the para-venous efflux of ISF.

As a channel protein on the cell membrane that controls water transport, aquaporin plays an important role in the development of edema (14, 15). AQP4 is one of the most important subtypes in the CNS, plays a vital role in CSF and ISF homeostasis, and is mainly located in astrocytes and ependymal cells (16). AQP4 is highly expressed a lot in the foot process of astrocytes (blood–brain), in the glial boundary membrane (brain–subarachnoid), and between ependymal cells and subependymal astrocytes (CSF in the brain and ventricles), and regulates the distribution and balance of water in the brain (17–19). In a recent study, researchers discovered that ISF clearance in the thalamus of AQP4 knockout rats was significantly prolonged compared with that in normal rats (20). In glioma followed by the vasogenic brain edema model, AQP4-null mice showed a significantly greater increase in water in the brain and intracranial pressure (ICP) than that in wild-type mice (16, 21, 22). These findings suggest that the trans-molecular water transport mediated by AQP4 plays a crucial role in the outflow of CSF.

Glioma, especially glioblastoma, is one of the most common tumors in the CNS and has high malignancy and poor prognosis (23, 24). Even with conventional surgery, adjuvant radiotherapy, and chemotherapy, the mean overall survival remains at only 12–18 months (25, 26). Cerebral edema and increased ICP are the common complications of glioma. Elevated ICP in glioma is mostly caused by tumor mass, hemorrhage, or edema (27). The continuous increase in ICP can cause a decrease in cerebral blood flow, leading to insufficient blood supply to the brain, which results in a vicious circle of cerebral ischemia, hypoxia, and edema, and consequently brain swelling, respiratory and circulatory failure, cerebral herniation, and even death (28). Unlike other solid tumors, glioma is difficult to eliminate because of the blood–brain barrier, blood–tumor barrier, and high ICP, which restrict the penetration of chemotherapy drugs into the tumors. Although there are numerous ways to improve drug delivery, such as using nanomaterials to penetrate the blood–brain barrier or directly injecting drugs into the tumor, progress has been limited (29).

Recent studies have confirmed three outflow pathways of CSF in healthy mice and rats (2, 3, 6–8, 30–32). However, in the glioma model, CSF drainage along the exiting cranial nerves and downstream lymph nodes was shown to be reduced (7). Both the deep cervical lymph nodes and the superficial cervical lymph nodes showed pathological changes, which indicated that the CSF in the cranial cavity and the normal outflow pathway from the cranial cavity were obstructed (7, 33). The dynamic balance between the production and drainage of CSF is an important element of maintaining stable ICP. Interruption in secretion or drainage of CSF leads to an increase in ICP (34). This may also explain why glioma patients exhibit symptoms of intracranial hypertension. Moreover, reports have shown that AQP4 is upregulated in glioma (35, 36), which indirectly indicates poor CSF outflow in glioma. In this study, we used *in vivo* magnetic resonance imaging (MRI) to observe dynamic changes in CSF drainage in a rat glioma model. In addition, we used Evans Blue *ex vivo* to evaluate the contribution of AQP4-mediated fluid flux to the movement of subarachnoid CSF into and through the brain parenchyma and AQP4 expression on the endfeet of astrocytes around the vessels in glioma using immunofluorescence.

## Materials and Methods

### Cell Culture

Rat glioma C6 cells were purchased from Shanghai Fuheng Cell Center and were cultured in F12K (Shanghai Fuheng Cell Center, Shanghai, China). All of the media were supplemented with 10% fetal bovine serum and 2% streptomycin-penicillin. Cells were maintained in the medium in an air atmosphere with 5% CO_2_ at 37°C.

### Animals

The animal study was reviewed and approved by the Institutional Animal Care and Use Committee of Wuhan University (E2020072803). Six-week male Sprague–Dawley rats (weight, 200–230 g) were purchased from Beijing Huafukang Bioscience Co., Ltd. (Beijing, China) and housed under specific pathogen−free conditions (60% relative humidity; 25°C room temperature) with a 12-h light/dark cycle. Animals were provided and maintained with food and water.

### Anesthesia

For all experiments, animals were anesthetized with 6% oxygen flow and 4% isoflurane (RWD Life Science Co., Ltd., Shenzhen, China), and anesthesia was maintained with 4% oxygen flow and 3% isoflurane.

### Tumor Model

Rats were fixed in a stereotaxic instrument (RWD Life Science Co., Ltd., Shenzhen, China); body temperature was maintained used a heating pad (37°C). The skull was thinned with a 1-mm diameter dental drill (RWD Life Science Co., Ltd., Shenzhen, China) at a location 3 mm lateral to the sagittal suture and 1 mm anterior of the bregma. Tumor cells at a density of 1.0 × 10^6^ cells/10 μl were extracted using a microinjection syringe pump (Beijing Zhongshi Dichuang Technology Development Co., Ltd., Beijing, China) at a speed of 2 μl/min for 2 min with a 25-μl GaoGe microliter syringe and circulating five times, which resulted in a total injected volume of 10 μl into the right striatum at a depth of 4.5 mm from the skull surface. The syringe was retracted 1 mm for 2 min and completely removed within 10 min while monitoring for any significant backflow occurred. The injection hole was closed with bone wax, and the scalp was sutured. The rats were observed in a cage with a 37°C heating pad until fully recovered from anesthesia.

### Intracisternal Injections

While rats were deeply anaesthetized and breathing steadily, the fur around the posterior end of the head was shaved to expose the skin. Rats were then fixed onto a stereotaxic instrument, and body temperature was maintained at 37°C using a heating pad. A skin incision was made over the occipital bone, and the covering muscle layers were carefully dissected to expose the atlanto-occipital membrane that covered the cisterna magna (37). A 30G injection inner tube (RWD Life Science Co., Ltd., Shenzhen, China) with a diameter of 15 cm PE10 tube (RWD Life Science Co., Ltd., Shenzhen, China) was used to penetrate the membrane perpendicular to the dura until resistance was overcome, which indicated entry into the cisterna magna. For the magnetic resonance imaging (MRI) experiments, 5 µl of Gadobutrol (MW ~1 kDa, Bayer Healthcare Co., Ltd., Beijing, China) was infused at a rate of 2 µl/min. For the *ex vivo* experiments, 5 µl of 2% Evans Blue (MW ~1 kDa, Macklin) was infused at a rate of 2 µl/min. After the infusion, the inner tube was left in place for 5 min, and tissue glue (3M Vetbond, 3M Animal care products) was applied to the injection site.

### ICP Monitoring of the Brain

Twelve days after implanting the tumor, the effect of tracer infusion on ICP was evaluated using an ICP microsensor (Codman Microsensor, Codman & Shurtleff Inc, Raynham, MA). An ICP cannula was inserted stereotactically into the right lateral ventricle, and the animal was allowed to equilibrate for 30 min. In the first group of animals, 5 µl of tracers were injected at 2 µl/min *via* the cisterna magna, and the ICP was measured for 60 min. In the second group, 5 µl of tracers was injected at different infusion rates of 4, 6, and 8 µl/min, and the ICP changes were observed. The ICP was recorded every 5 min (38, 39).

### MRI of CSF Dynamics

Twelve days after implanting the tumor, glioma-bearing rats were fixed in an eight-channel rat coil (Suzhou Medcoil Healthcare Co., Ltd., Suzhou, China). Data were acquired on a 3T MRI system (Prisma, Siemens, Germany). To measure the contrast agent distribution after cisterna magna infusion, rats were injected with 5 µl Gadobutrol at a rate of 2 µl/min *via* the cisterna magna and scanned 0.5, 1, 2, 3, and 4 h after Gadobutrol administration. T1-weighted, T2-weighted, and T1-mapping image sequences were acquired. T1-weighted image parameters were echo time/repetition time = 14/500 ms; field of view (FOV), 50 × 50 mm; slice thickness = 0.8 mm; and 22 slices. T2-weighted image parameters were echo time/repetition time = 30/4,150 ms; FOV, 50 × 50 mm; slice thickness = 0.8 mm; and 22 slices. T1-mapping image parameters were echo time/repetition time = 3.5/12 ms; FOV, 50 × 50 mm, slice thickness = 0.5 mm, and 60 slices. Signal changes were measured on the T1 mapping images over time in regional tissues. The T1-weighted MRIs were used to anatomically guide the placement of the regions of interest (ROIs). One slice of the T1-weighted image corresponded to three slices of the T1 mapping image. The sizes of the ROIs were 6.5 mm × 6.8 mm. ROIs were drawn on six coronal slices of the MRI image and comprised the striatum, lateral ventricle, third ventricle, fourth ventricle, olfactory bulb, and pineal recess. The left striatum ROI was taken from the symmetrical right striatum or the symmetrical right tumor area (ROIs shown in **Supplementary Figure S1**). The signals from each of the anatomical ROIs were measured using Horos software (Horos version 3.3.6, Horos Technologies Ltd.).

### Glymphatic-Mediated Evans Blue Distribution

After injection of 5 µl 2% Evans Blue into the cisterna magna, rats were maintained under 3% isoflurane. After 0.5, 1, 2, 3, or 4 h, the rats were euthanatized by isoflurane and were perfused with 4% paraformaldehyde for cardiac perfusion. Brains were then removed and stored at 4°C in the dark (13).

After being injected with 2% Evans Blue at 4 h, the brains were sectioned at 10-μm thickness in the coronal plane using a freezing microtome (Leica CM 1950, Leica Biosystems, Germany) and collected on labeled gelatin slides. Slices were blocked in 3% bovine serum albumin (Servicebio Cat# G5001) for 0.5 h at room temperature, incubated with a primary antibody overnight at 4°C, and incubated with a secondary antibody for 10 min at room temperature. After washing, all slices were incubated with 4′,6-diamidino-2-phenylindole (DAPI, Servicebio Cat# G1012) for 10 min at room temperature and mounted using an anti-fade mounting medium. Primary antibodies included anti-glial fibrillary acidic protein (GFAP, Servicebio Cat# GB12096, 1:500) and anti-astrocytic aquaporin 4 (AQP4, Servicebio Cat# GB11529, 1:500). Secondary antibodies included Alexa Fluor 488-conjugated goat anti-mouse (Servicebio Cat# GB25301, 1:400) and Cy3-conjugated goat anti-rabbit (Servicebio Cat# GB21303, 1:300). Evans Blue was acquired with an excitation filter set at 600 nm and an emission filter set at 700 nm (13). Slices were viewed using a confocal microscopy (Leica sp8, Leica Biosystems, Germany).

### Histology and Immunohistochemical/Immunofluorescence Staining

Brains removed from the rats were fixed in 4% paraformaldehyde for 24 h and embedded in paraffin after ethanol dehydration. Microtome sections (4-µm thick) after deparaffinization and rehydration were used in hematoxylin and eosin (H&E), and Ki-67 immunohistochemical and immunofluorescence staining, following antigen retrieval in citrate buffer (pH 6) for 25 min. For immunofluorescence staining, slices were blocked in 3% bovine serum albumin for 0.5 h at room temperature, incubated with a primary antibody overnight at 4°C, and incubated with a secondary antibody for 10 min at room temperature. After washing, all slices were incubated with DAPI for 10 min at room temperature and mounted using an anti-fade mounting medium. Primary antibodies included anti-GFAP mouse mAb, anti-alpha smooth muscle actin (α-SMA, Servicebio Cat# GB111364, 1:5000), cluster designation 34 recombinant rabbit monoclonal antibody (CD34, HUABIO Cat# ET1606-11, 1:3,000), and anti-AQP4. Secondary antibodies included Alexa Fluor 488-conjugated goat anti-mouse, fluorescein isothiocyanate (FITC)-conjugated goat anti-rabbit (Servicebio Cat# GB22303, 1:500), Cy3-conjugated goat anti-rabbit, and Cy5-conjugated goat anti-rabbit (Servicebio Cat# GB27303, 1:300).

Slices were imaged at low power resolution using confocal microscopy. To quantify GFAP, α-SMA, CD34, and AQP4 expression in the fixed slices, exposure levels were determined according to normal brain slices and maintained constant throughout the study. The ROI used for quantitative fluorometry encompassed the right striatum.

### Statistics Analysis

Statistical analysis was performed using SPSS 23.0 (IBM Corp.). Data are presented as means ± standard deviations (SDs). An independent-samples t-test was used to compared ICP between the two groups. Paired-samples t-tests were used to compare ICP between each time point in each group. Mann–Whitney U test was used to compare MRI measures and average fluorescent intensity between the two groups. For comparisons of the adjacent time points of MRI data within each group, Wilcoxon’s signed-rank test was used. A two-sided p-value <0.05 was considered as statistically significant.

## Results

### Effect of Infusion Rate on ICP

Previously, it was reported that the cerebroventricular injection of a high doses of gadolinium in animals can induce neurotoxic effects, such as focal seizure activity, ataxia, and delayed tremor (40, 41). To prevent neurotoxicity from a large amount of gadolinium accumulating in the brain in a short time and intracranial hypertension caused by the rapid infusion rate into the cisterna magna (42), we measured ICP at different infusion rates to ensure a safe infusion rate.

**Figure 1:**
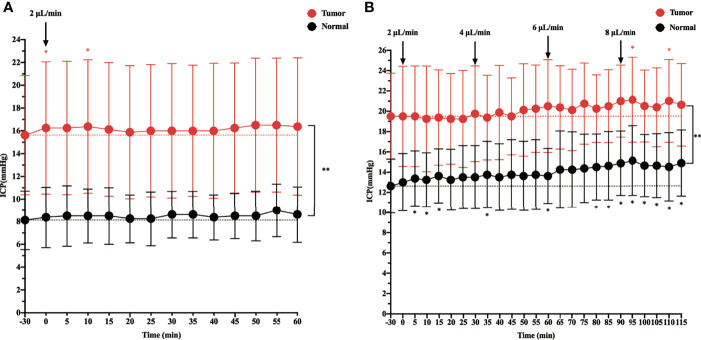
The effect of intracisternal tracer infusion on intracranial pressure (ICP). Ventricular ICP was monitored continuously during infusion of CSF tracer Gadobutrol in rats. The dotted line represents the baseline, which is the “-30 min” timepoint. The dots represent the recorded ICP of each timepoint. The branches represent standard error. **(A)** The ICP was measured 60 min at the rate of 2 µl/min intracisternal infusion of Gadobutrol. **(B)** The ICP was measured by increasing the infusion rates of 2, 4, 6, and 8 µl/min (*p < 0.05, the ICP after injected compared with the baseline in the normal; *p < 0.05, the ICP after injected compared with the baseline in the tumor; **p < 0.05, the ICP in the tumor compared with the normal at each time point, n = 8 per group).

Before starting the infusion, we recorded the baseline physiological ICP, which was 8.125 mmHg in the control group and 15.625 mmHg in the tumor group. When ICP was measured in rats during intracisternal Gadobutrol infusion, the control group did not show a significant change in ICP at the rate of 2 µl/min for 60 min (**Figure 1A**). In the tumor group, apart from ICP slightly increasing at 0 and 10 min after the infusion, there was no significant change at other time points. In addition, the overall ICP of the tumor group was higher than that of the control group.

When the infusion rate was increased to 4, 6, and 8 µl/min, a progressive significant elevation in ICP was noted in the control group. Specifically, when the control group was injected at a rate of 8 µl/min, the ICP at each time point was higher than that at baseline. In the tumor group, although the ICP gradually increased at 4 and 6 µl/min, there was no significant change from baseline. However, at 5 and 25 min after the 8 µl/min infusion, there was a significant change in ICP from baseline, and there was a trend elevation at other time points, although this was not significant. In addition, the overall ICP of the tumor group was higher than that of the control group at different infusion rates (**Figure 1B**).

### Intracisternal Gadobutrol Clearance in the Glioma-Bearing Rat

**Figure 2:**
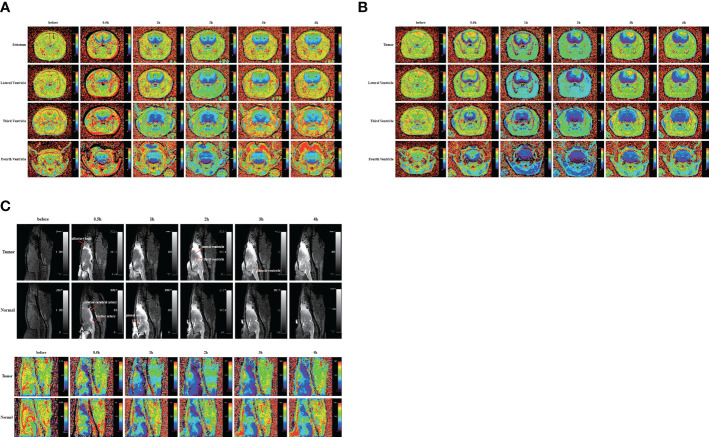
Gadobutrol distribution in the different parts of the brain in 0.5, 1, 2, 3, and 4 h. **(A)** The T1−mapping images in the coronal plane of the normal group. **(B)** The T1−mapping images in the coronal plane of the tumor group. **(C)** The T1-weighted images and the T1−mapping images in the sagittal plane of the tumor and normal groups. The black circles represent regions of interest (n = 9 per group).

**Figure 3:**
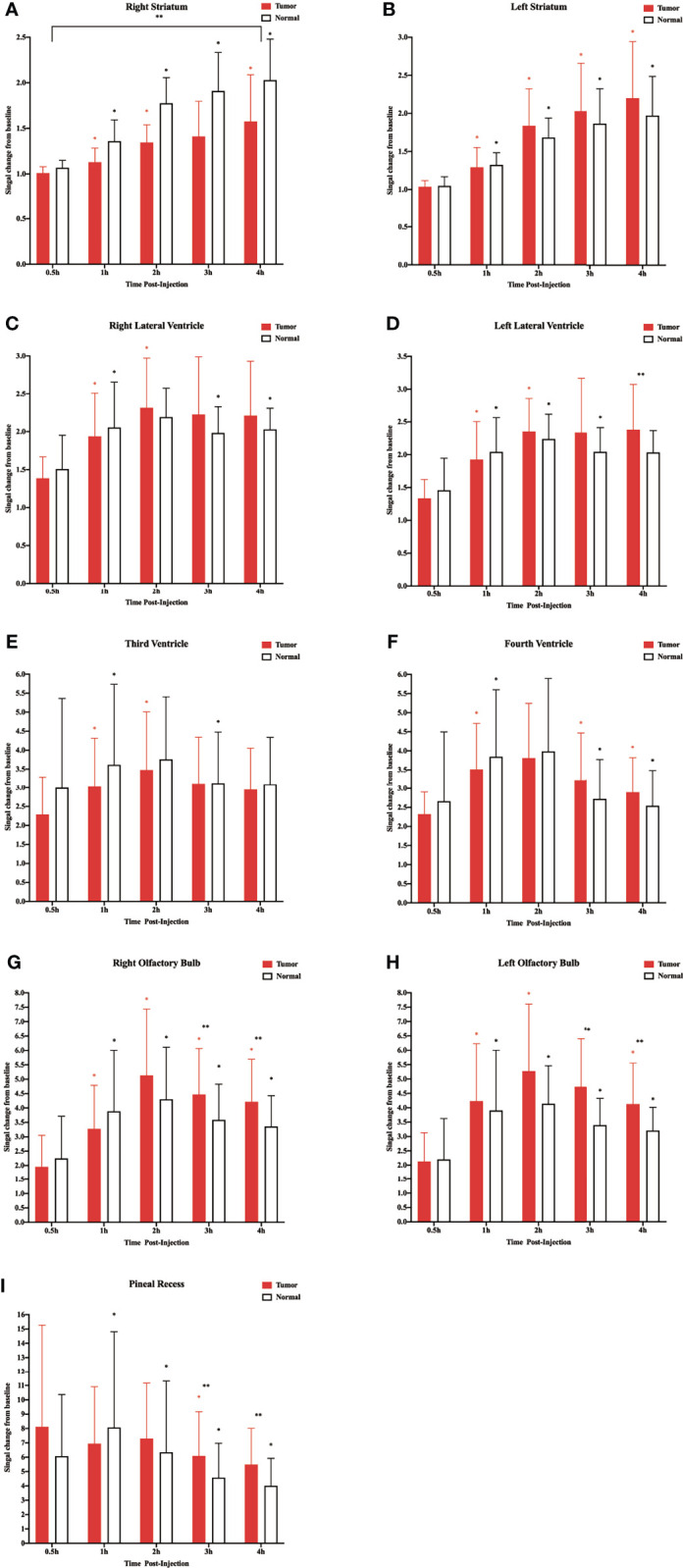
Corresponding quantification of the signal intensity in the different regions of interest. **(A)** Right striatum. **(B)** Left striatum. **(C)** Right lateral ventricle. **(D)** Left lateral ventricle. **(E)** Third ventricle. **(F)** Fourth ventricle. **(G)** Right olfactory bulb. **(H)** Left olfactory bulb. **(I)** Pineal recess (*p < 0.05, the signal intensity compared with adjacent time points within the normal group; *p < 0.05, the signal intensity compared with adjacent time points within the tumor group; **p < 0.05, the signal intensity in the tumor compared with the normal at each time point, n = 9 per group).

In the above experiment, we determined that 2 µl/min was a safe injection speed that did not affect the change in ICP. Next, to investigate whether there is a change in CSF outflow through the glymphatic system in the presence of a glioma, MRI was used to measure the contrast agent intensity at the cisterna magna over time (**Figures 2A, B**). In the glioma-bearing rats, contrast-enhanced MRI was performed 12 days post-inoculation (**Supplementary Figure S2**). In the striatum, the contrast agent intensity continuously increased in both the tumor and control groups. In the right striatum with the glioma, the signal intensity at each time point was clearly lower than that in the control group after the injection. In the tumor group, signal intensities differed significantly between 0.5 and 1 h, 1 and 2 h, and 3 and 4 h. In the control group, signal intensities between adjacent time points were significantly different (**Figure 3A**). In the left striatum, signal intensities between adjacent time points within each group were significantly different (**Figure 3B**). Comparison between the contralateral and ipsilateral striatum in the two groups showed no difference in the signals of the contralateral and ipsilateral striatum in the control group (**Supplementary Figure S3B**). In contrast, in the tumor group, the signal of the right tumor was lower than that of the contralateral striatum from 1 to 4 h (**Supplementary Figure S3A**).

In the two lateral ventricles, signal intensities were significantly different between 0.5 and 1 h, and 1 and 2 h in the tumor group. Furthermore, comparisons of signal intensities between adjacent time points within the control group showed that the signal intensity of the right lateral ventricle differed significantly among all time points except between 1 and 2 h, and that of the left lateral ventricle differed significantly among all time points between 3 and 4 h. In the left lateral ventricle, the signal intensity of the tumor group was higher than that of the control group at 4 h after injection (**Figures 3C, D**).

In the third and fourth ventricles, the signal intensity of the two groups reached a peak at 2 h. In the third ventricle, signal intensities were significantly different between 0.5 and 1 h, and 1 and 2 h in the tumor group. In the control group, signal intensities were significantly different between 0.5 and 1 h, and 2 and 3 h (**Figure 3E**). In the fourth ventricle, signal intensities differed significantly among all time points except between 1 and 2 h in the two groups (**Figure 3F**).

The sagittal MRI data revealed that the two groups of contrast agents gathered in the brain parenchyma in close vicinity to the ventral basal cisterns 0.5 h after the injection and was obvious around the basilar and anterior cerebral arteries. Subsequently, signals of the olfactory bulbs and the pineal recess also changed between 1 and 4 h (**Figure 2C**). The signal intensity of the bilateral olfactory bulbs of the two groups reached a peak at 2 h. In the bilateral olfactory bulbs, the signal intensity of the tumor group was higher than that of the control group at 3 and 4 h. In the bilateral olfactory bulbs of the control group, signal intensities between adjacent time points differed significantly. In the right olfactory bulb of the tumor group, signal intensities between adjacent time points differed significantly. In the left olfactory bulb of the tumor group, signal intensities differed significantly between 0.5 and 1 h, 1 and 2 h, and 3 and 4 h (**Figures 3G, H**). In the pineal recess, the signal intensity was significantly higher in the tumor group than that in the control group at 3 and 4 h. The signal intensity differed significantly between 2 and 3 h in the tumor group, and signal intensities between adjacent time points differed significantly in the control group (**Figure 3I**).

### Evans Blue Outflow in Glioma-Bearing Rat

**Figure 4:**
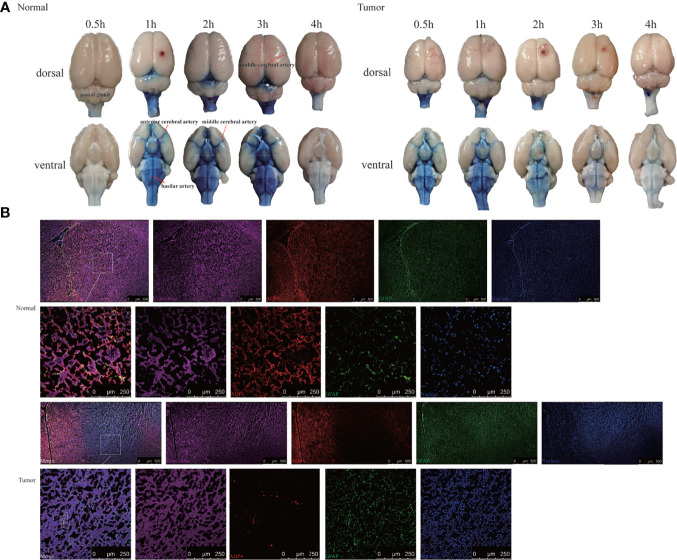
Evans Blue distribution after the cisterna magna injection. **(A)** The bright field of the whole brain after injection of Evans Blue at each time point. Left: the normal group; right: the tumor group (n = 6 per time point). **(B)** *In vitro* assessment of the contribution of AQP4 in CSF tracer influx into the brain parenchyma 4 h after injection. Upper: the normal group; down: the tumor group. The first and third rows: scale bar, 500 μm; the second and fourth rows: scale bar, 250 μm (Evans Blue, purple; AQP4, red; GFAP, green; merge, white circles). GFAP, glial fibrillary acidic protein (astrocytic marker); AQP4, aquaporin 4 (n = 3 per group).

To confirm the results using another imaging method, we performed bright field photography of the brain (*ex vivo*) at 0.5, 1, 2, 3, and 4 h after intracisternal injection of Evans Blue (**Figure 4A**). In the control group, Evans Blue was distributed clearly in the pineal recess, basal cisterna, and surrounding the circle of Willis at 1–3 h. In the tumor group, the distribution time of Evans Blue was earlier: the pineal recess, basal cisterna, and surrounding the circle of Willis distributions were obvious at 0.5 h, which subsequently gradually weakened. Regardless of whether observations of the brain were made dorsally or ventrally, the distribution of Evans Blue around the middle cerebral artery was not obvious in the tumor area compared with that in the contralateral side. Overall, the tumor group had less Evans Blue in the pineal recess, basal cisterna, and surrounding the circle of Willis than did the control group. In addition, Evans Blue in the ventral side of the brain was decreased slowly in the tumor group, whereas, the decrease in the control group was obvious from 3 to 4 h. Moreover, the changes in the pineal recess in the tumor group were not as obvious as those in the control group.

This led us to question why Evans Blue decreased around the tumor area and whether it was related to AQP4 on the foot process of astrocytes. To test this, CSF tracer influx in the brain parenchyma was observed by labeling astrocytes and AQP4. Confocal images showed that Evans Blue moved into the brain parenchyma and distributed in the ISF in the control group. Moreover, AQP4 was detected around GFAP-labeled astrocytes, and high-power resolution showed that Evans Blue was distributed around AQP4. In the tumor group, the fluorescence intensity of GFAP in the tumor area was significantly higher, whereas the fluorescence intensity of AQP4 was significantly weaker. The fluorescence intensity of Evans Blue in the tumor area was also reduced. Using high-power resolution, we found that AQP4 around the GFAP-labeled astrocyte was decreased, and Evans Blue surrounding AQP4 was also decreased (**Figure 4B**).

### Effect of AQP4 on CSF Flux in the Parenchyma

**Figure 5:**
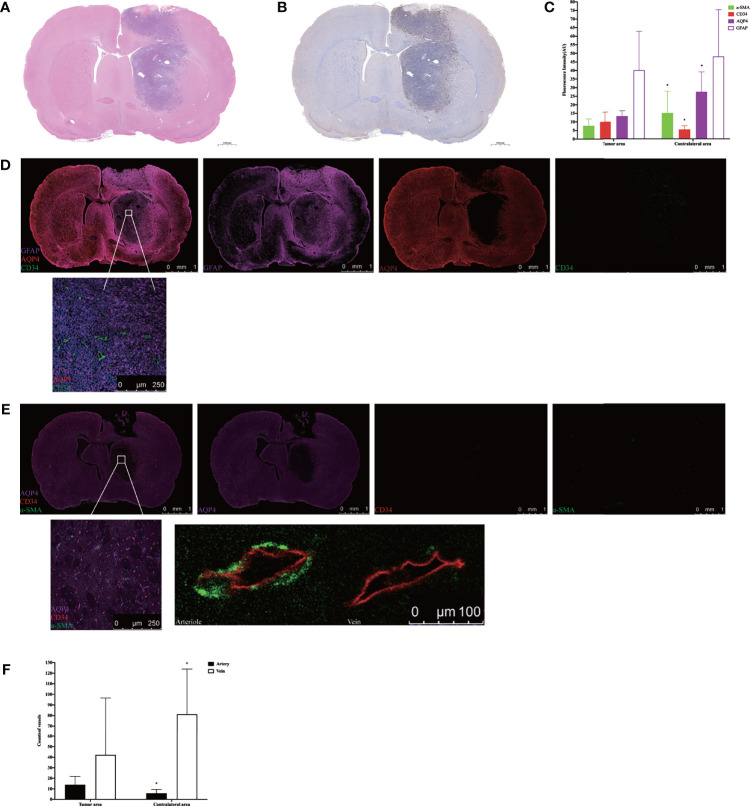
Histology and immunohistochemical/immunofluorescence staining. **(A)** H&E staining in the tumor tissue. **(B)** Ki-67 immunohistochemical staining in the tumor tissue. **(C)** The mean fluorescence intensity of GFAP, α-SMA, CD34, and AQP4 in the tumor area compared with the contralateral area (*p < 0.05. n = 8 per group). **(D)** The positional relationship between GFAP, CD34, and AQP4. Upper: scale bar, 1 mm; down: scale bar, 250 μm (GFAP, purple; AQP4, red; CD34, green) (n = 8). **(E)** The AQP4 expression in the perivascular space in the tumor tissue. The endothelium of all blood vessels was labeled with red, while vascular pericytes were labeled with green. The arteries and arterioles were labeled with both green and red, while veins were labeled with only red. Upper: scale bar, 1 mm; down left: scale bar, 250 μm; down right: scale bar, 100 μm (AQP4, purple; CD34, red; α-SMA, green) (n = 8). **(F)** The number of CD34^+^α-SMA^+^ arteries and CD34^+^α-SMA^−^ veins in the tumor area compared with the contralateral area (*p < 0.05, n = 8 per group). GFAP, glial fibrillary acidic protein (astrocytic marker); α-SMA, alpha smooth muscle actin (vascular pericytes marker); CD34, cluster designation 34 (vascular endothelial cells marker); AQP4, aquaporin 4.

H&E staining showed that nuclei were abundant in the tumor area (**Figure 5A**), and immunohistochemistry revealed greater expression of Ki-67 in the tumor area than the contralateral area in the tumor tissue (**Figure 5B**). The subsequent quantification of GFAP, α-SMA, CD34, and AQP4 expression levels in tumor tissue using immunofluorescence showed that the expression of α-SMA, AQP4, and GFAP in the tumor area was lower than that in the contralateral area, whereas the expression of CD34 was higher than in the contralateral area (**Figure 5C**). Using high power resolution in the tumor area, we observed that GFAP expression was lower in the internal core of the tumor than in the peripheral area, CD34-positive vessels were surrounded by GFAP-positive astrocytes, and AQP4 was weakly expressed (**Figure 5D** and **Supplementary Figure S4**). The slices were labeled with α-SMA and CD34 simultaneously, which allowed arteries to be distinguished from veins, thereby observing the AQP4 expression in the peri-arterial and the peri-venous spaces. In the two areas, data indicated that the number of CD34^+^α-SMA^−^ veins was more than that of CD34^+^α-SMA^+^ arteries. Moreover, the number of CD34^+^α-SMA^+^ arteries in the tumor area were more than in the contralateral area. In contrast, the number of CD34^+^α-SMA^−^ veins in the tumor area were less than in the contralateral area. In the tumor area, irrespective of arteries or veins, AQP4 expression surrounding the vessels was weak (**Figures 5E, F**).

## Discussion

**Figure 6:**
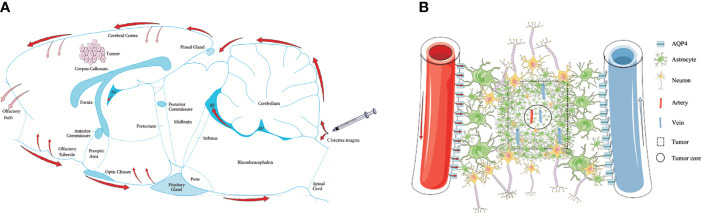
Schematic of the glymphatic system alternations in glioma. **(A)** Through cisterna magna injection, a part of the CSF tracers refluxed into each ventricle; the other entered the cerebral cortex through the subarachnoid space (red arrows). The main perineural egress site is along the olfactory nerve through the cribriform plate, in which the tumor was blocked (reddish arrows). The CSF drainage from the cerebral cortex into the tumor was blocked (reddish arrows). **(B)** In the tumor area, the GFAP and AQP4 expression in the internal core was lower than in the periphery. In the tumor area, irrespective of arteries or veins, AQP4 expression surrounding the vessels was weak.

Three CSF outflow pathways conclude through the subarachnoid space, into the venous sinuses and downstream lymph nodes, along the peripheral nerves to the cervical and spinal cord lymph nodes, and into the glymphatic system. In this study, we used *in vivo* MRI and *ex vivo* Evans Blue recording for 4 h to observe the outflow pathway of CSF. By injecting *via* the cisterna magna in control rats, CSF first flowed into the vicinity of the brain surface, where a proportion of CSF that flowed through the subarachnoid space moved into the venous sinuses. Some of the CSF flowed forward to reach the olfactory bulbs and along the peripheral nerves to the cervical and spinal cord lymph nodes. Another portion of the CSF flowed through the subarachnoid space and entered into the brain parenchyma from the para-vascular spaces of the arteries. However, in glioma-bearing rats, the images showed that CSF tracers gathered near both olfactory bulbs. In the bilateral olfactory bulbs, the signal intensity of the tumor group was higher than that of the control group at 3 and 4 h. Most fibers of the olfactory nerve terminate in the olfactory bulb; thus, CSF can be transported along the side of the olfactory artery and into the olfactory bulb (43). These findings indirectly indicated that the circulation of CSF was hindered in glioma reflected by the reduction in signal along the exiting cranial nerves, which is consistent with the previous data (33). Second, through MRI and *ex vivo* Evans Blue bright field, we revealed that the tracer signal in the pineal recess reduced slowly, which indicated that the tumor hindered the outflow of CSF from the venous sinuses to the downstream lymph nodes (**Figure 6A**). These results are consistent with a previous study (33).

Through cisterna magna injection, a part of the tracers refluxed into each ventricle. In the lateral ventricles, we observed that the signal of the tumor group was lower than that of the control group at 0.5 and 1 h, which may be attributed to the compression of the tumor. Subsequently, the signal in the lateral ventricles of the tumor group decreased slowly, which resulted in the overall signal of the third ventricle being lower than that of the control group. The slow outflow of tracers in the lateral ventricles may have been due to the compression of the tumor on the right lateral ventricle, which would narrow the drainage route. However, CSF production is reduced with high ICP (44, 45). According to the anatomical structure, the lateral ventricles are connected through the interventricular foramen, and the left lateral ventricle produces CSF normally, while the two sides help each other to dilute the tracers in the lateral ventricles. However, CSF production was lower in the tumor group than in the control group; thus, the tracer dilution in the lateral ventricles was lower, which led to a slow decrease in signal. The signal of the fourth ventricle of the tumor group was higher than that of the control group at 3 and 4 h, partly because of the increased ICP resulting in tracers amassing there. The other reason was shunted to the subarachnoid space, and the CSF outflow from the venous sinuses to the lymph nodes was blocked.

In addition to the reflux into each ventricle, some of the tracers moved into the brain parenchyma through the subarachnoid space. In the right striatum, the transport of tracers to the tumor was delayed and distributed in the peripheral region of the tumor, and ICP was shown to be high in the right lateral ventricle, showing that the tumor had high pressure. We speculated that the pressure of the tumor was greater than that of the surrounding tissues, which prevented the tracer from entering the tumor area. Moreover, this showed that there were more tracers in the contralateral area than in the tumor area, which indicated compensatory tumor regulation. Because the route to the tumor area was blocked, outflow changed to the healthy side. This supports the notion that the para-arterial influx of subarachnoid CSF is limited in glioma, which was confirmed around the middle cerebral artery in the Evans Blue distribution.

CSF from the subarachnoid space flows through the para-arterial space into the brain interstitium, and pericytes rather than endothelial cells have been reported to be primary cells that constitute mature vessels (46, 47). There were more CD34-positive endothelial cells and fewer α-SMA-positive cells in the tumor area than in the contralateral area, which suggested that there were many immature vessels. Studies have shown that vessel maturity is related to the delivery of chemotherapeutic drugs (48). The low maturity of vessels in the tumor may also affect the CSF influx.

AQP4 is a key protein in the glymphatic system and promotes the flow of CSF from the peri-arterial space into the cerebral interstitium to exchanges of CSF with ISF. Although the immunofluorescence revealed that the number of CD34^+^α-SMA^+^ arteries in the tumor area was more than in the contralateral area, the expression of AQP4 in the astrocytes around the vessels was weak in the tumor area. These results indicate that the para-arterial influx of subarachnoid CSF is limited in glioma. Owing to the loss of AQP4 in the tumor area, the exchange of CSF and ISF is decreased and the clearance of harmful solutes is limited in tumors, which has been confirmed in both Alzheimer’s disease and Parkinson’s disease (30, 49–51). Furthermore, it has been reported that CSF can transport CNS-derived antigens to the dural sinuses to be captured by T cells, which induces an immune response (52). Impaired CSF outflow in glioma affects the activation and proliferation of anti-tumor T cells, which provides a favorable environment for the progression of glioma. Moreover, the number of CD34^+^α-SMA^−^ veins in the tumor area was less than in the contralateral area, which indicated that the para-venous efflux of ISF was lower in the glioma (**Figure 6B**).

We also observed an interesting phenomenon: GFAP and AQP4 expression in the internal core and periphery of glioma differed, where the peripheral area was significantly enriched with GFAP and AQP4. Studies have demonstrated that the core is characterized by high hypoxic and low proliferation indices, whereas the periphery contains a more vascularized and more oxygenated area (53). Whether this is related to the formation of edema around the tumor requires further exploration.

There are several limitations to the current study. We did not include a sham-operated group because the surgery itself can induce problems in CSF circulation. In traumatic brain injury studies, sham-treated mice are anesthetized, hung from the string, and allowed to fall and recover. Such data indicate that the glymphatic pathway is not affected, and AQP4 localization is polarized to the perivascular astrocytic endfeet surrounding the cerebral microcirculation (54). Thus, we expect that the sham-operated groups would not show any difference from the control group. Second, we used a 3-T MRI for scanning, which does not allow the optic nerve to be distinguished from the olfactory nerve. It is difficult to quantitatively analyze the contrast intensities of the cranial nerves. Therefore, direct observations of the changes in CSF outflow through the peripheral nerves were not possible. Finally, further explorations are needed to determine the mechanisms of the affected ventricles in glioma.

In summary, the glymphatic system is a perivascular pathway mediated by AQP4 and is mainly composed of three parts: the arterial perivascular space pathway, the CSF-ISF exchange, and the venous perivascular space pathway. In this study, we found that glioma blocked the arterial perivascular space pathway of the glymphatic system and specifically reduced the key protein, AQP4. These findings suggest that the decreased AQP4 in glioma may be related to the reduced transportation of drugs to the tumor area when administered *via* intrathecal injection. Using AQP4 as a medium may restore the glymphatic system of the glioma and improve drug delivery.

## Data Availability Statement

The original contributions presented in the study are included in the article/**Supplementary Material**. Further inquiries can be directed to the corresponding author.

## Ethics Statement

The animal study was reviewed and approved by Institutional Animal Care and Use Committee of Wuhan University.

## Author Contributions

DX completed the whole experiment and wrote the initial draft of the paper. DX made the glioma rat model assisted by JZ. HM and WBS helped in adjusting the MRI parameters. HL provided the suggestions in the initial draft. HBX conceived the idea of the study and provided the funding. All authors contributed to the article and approved the submitted version.

## Funding

This work was financially supported by the National Key Research and Development Plan of China (Project 2017YFC0108803). Translational Medicine and Interdisciplinary Research Joint Fund of Zhongnan Hospital of Wuhan University (Grant No. ZNJC201911), and National Natural Science Foundation of China (Grant No. 81801667).

## Conflict of Interest

The authors declare that the research was conducted in the absence of any commercial or financial relationships that could be construed as a potential conflict of interest.

## Publisher’s Note

All claims expressed in this article are solely those of the authors and do not necessarily represent those of their affiliated organizations, or those of the publisher, the editors and the reviewers. Any product that may be evaluated in this article, or claim that may be made by its manufacturer, is not guaranteed or endorsed by the publisher.

## Supplementary Material

Supplementary Figure 1 Regions of interest for signal intensity calculation. **(A)** Striatum in the normal group. **(B)** Striatum in the tumor group. **(C)** Lateral ventricles. **(D)** Third ventricle. **(E)** Fourth ventricle. **(F)** Olfactory bulb. **(G)** Pineal recess. The black circles represent regions of interest.

Supplementary Figure 2 T2-weighted image for glioma size on twelve days after C6 cells implantation. The yellow line indicated the margin of the glioma.

Supplementary Figure 3 Corresponding quantification of the signal intensity in the contralateral and ipsilateral striatum in each group. **(A)** Compare the contralateral striatum and the right tumor in the tumor group. **(B)** Compare the contralateral and ipsilateral striatum in the normal group. (**P<0.05 The signal intensity in the tumor compared with the normal at each time point in the tumor group. n=9 per group).

Supplementary Figure 4 The entire tumor under the 10x microscope. The star represented the internal core, the arrow represented the periphery. (GFAP purple; AQP4 red; CD34 green). (n = 8). GFAP: glial fibrillary acidic protein (astrocytic marker); CD34: cluster designation 34 (vascular endothelial cells marker); AQP4: aquaporin 4. Scale bar, 500 μm.
