# Supplementary figures and images for: Impediment of Cerebrospinal Fluid Drainage Through Glymphatic System in Glioma
Source: Front Oncol. 2022 Jan 10;11:790821. doi: 10.3389/fonc.2021.790821 (PMC8784869; doi:10.3389/fonc.2021.790821)

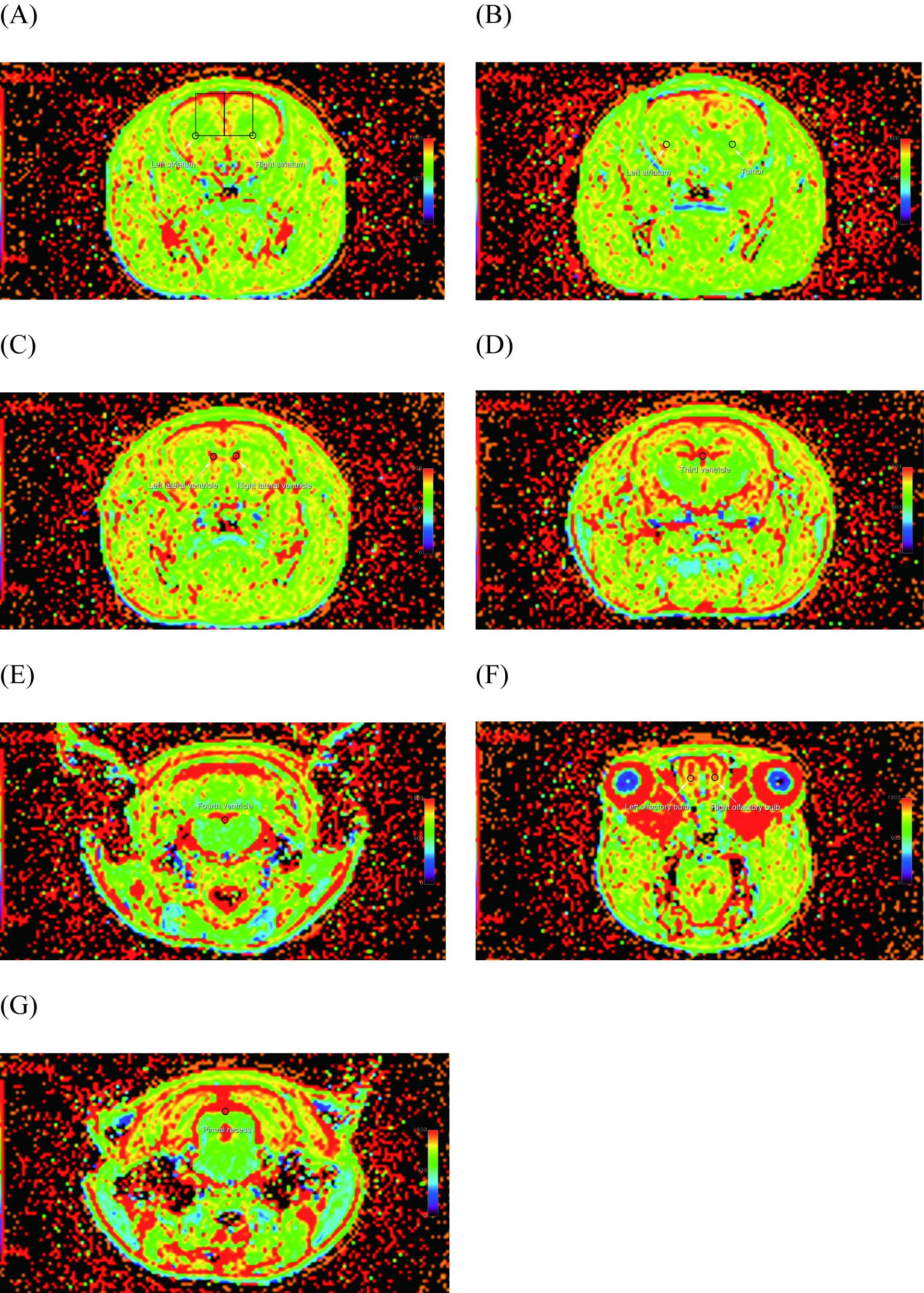

Supplement: Supplementary Figure 1 — Regions of interest for signal intensity calculation. (A) Striatum in the normal group. (B) Striatum in the tumor group. (C) Lateral ventricles. (D) Third ventricle. (E) Fourth ventricle. (F) Olfactory bulb. (G) Pineal recess. The black circles represent regions of interest. [file Image_1.tif]

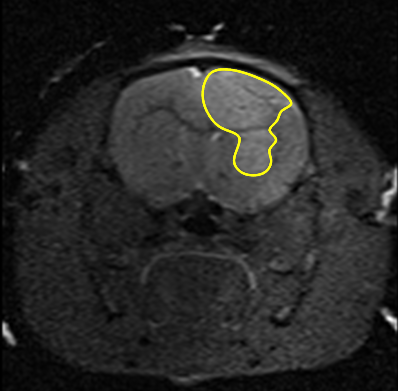

Supplement: Supplementary Figure 2 — T2-weighted image for glioma size on twelve days after C6 cells implantation. The yellow line indicated the margin of the glioma. [file Image_2.tif]

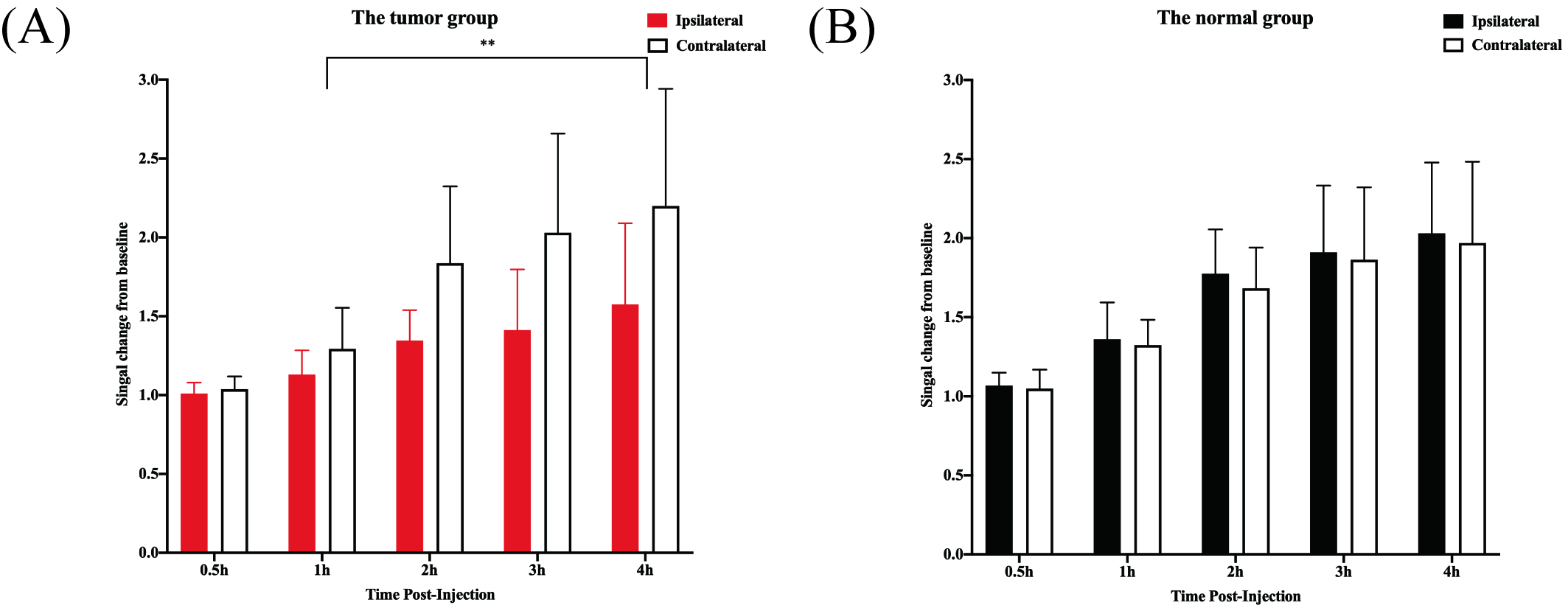

Supplement: Supplementary Figure 3 — Corresponding quantification of the signal intensity in the contralateral and ipsilateral striatum in each group. (A) Compare the contralateral striatum and the right tumor in the tumor group. (B) Compare the contralateral and ipsilateral striatum in the normal group. (**P<0.05 The signal intensity in the tumor compared with the normal at each time point in the tumor group. n=9 per group). [file Image_3.tif]

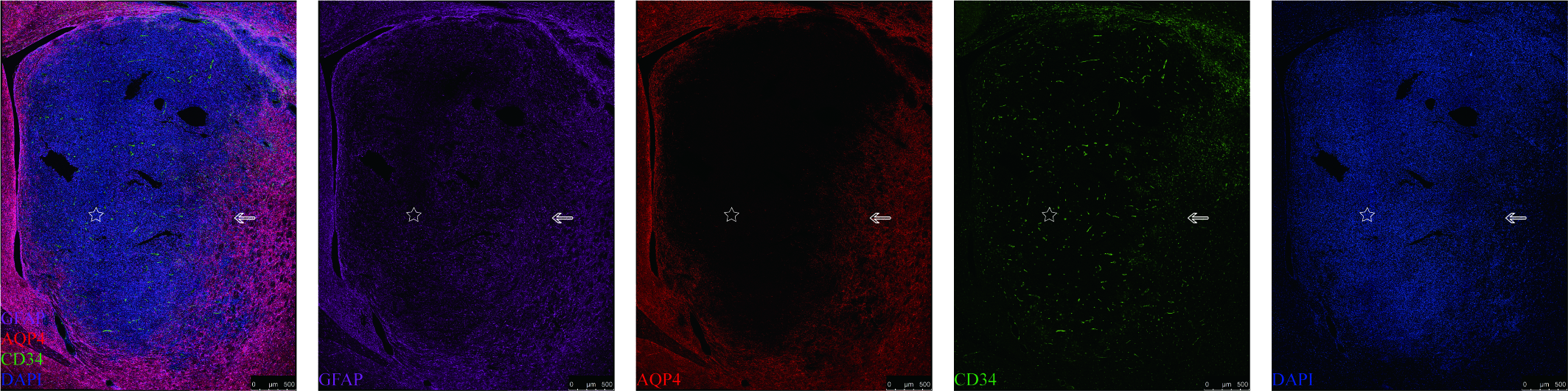

Supplement: Supplementary Figure 4 — The entire tumor under the 10x microscope. The star represented the internal core, the arrow represented the periphery. (GFAP purple; AQP4 red; CD34 green). (n = 8). GFAP: glial fibrillary acidic protein (astrocytic marker); CD34: cluster designation 34 (vascular endothelial cells marker); AQP4: aquaporin 4. Scale bar, 500 μm. [file Image_4.tif]
